# Elevated Ozone Deteriorates Grain Quality of *Japonica* Rice cv. Koshihikari, Even if it Does Not Cause Yield Reduction

**DOI:** 10.1186/s12284-016-0079-4

**Published:** 2016-02-24

**Authors:** Hiroko Sawada, Keita Tsukahara, Yoshihisa Kohno, Keitaro Suzuki, Nobuhiro Nagasawa, Masanori Tamaoki

**Affiliations:** Center for Environmental Biology and Ecosystem Studies, National Institute for Environmental Studies, Onogawa 16-2, Tsukuba, Ibaraki 305-8506 Japan; JSPS Research Fellow, Japan Society for the Promotion of Science, ᅟChiyoda-ku, Tokyo, 102-0083 ᅟJapan; Central Research Institute of Electric Power Industry, Abiko, Chiba 270-1194 Japan; NARO Institute of Crop Science, Tsukuba, Ibaraki 305-8518 Japan; Department of Agribusiness, Akita Prefectural University, Minami akita-gun, Akita 010-0444 Japan

**Keywords:** Grain Quality, Ozone Stress, Rice, Starch

## Abstract

**Background:**

It is becoming clear that ozone affects not only grain yield but also grain quality in rice. However, the biochemical mechanisms responsible for ozone-induced changes in appearance quality or components are poorly understood. We analyzed appearance quality and starch composition in the rice cultivars “Koshihikari” (*japonica*) and “Kasalath” (*indica*) grown under elevated ozone conditions.

**Results:**

Elevated ozone significantly increased the proportion of immature (mainly chalky) kernels in “Koshihikari” but not in “Kasalath”. Scanning electron microscopy of transverse sections of kernels showed that endosperm starch granules of “Koshihikari” ripened under elevated ozone were loosely packed with large spaces and contained irregular rounded granules. Amylose content was increased in “Koshihikari” kernels with ozone exposure, but was unchanged in “Kasalath” kernels. Distribution analysis of amylopectin chain length showed that ozone induces a decrease of long-side chains and alterations of short side-chains in “Koshihikari” kernels. Furthermore, *Starch Synthase (SS) IIIa* transcript levels in “Koshihikari” caryopses were decreased by elevated ozone.

**Conclusions:**

The *japonica* cultivar “Koshihikari” showed significant deterioration in appearance quality of kernels caused by abnormal starch accumulation due to exposure to ozone. The alteration patterns of amylose and amylopectin in ozone-exposed rice kernels are similar to those in rice kernels harvested from *SSIIIa*-deficient mutants. These findings suggest that the increase of chalky kernels in ozone-treated “Koshihikari” is partly attributable to the repressed expression of *SSIIIa* involved in amylopectin side-chain elongation with ozone exposure. Elevated ozone reduced appearance quality in “Koshihikari” although it did not impair starch properties contributing to the eating quality of cooked rice.

**Electronic supplementary material:**

The online version of this article (doi:10.1186/s12284-016-0079-4) contains supplementary material, which is available to authorized users.

## Background

Tropospheric ozone is a major gaseous pollutant generated by the photochemical reactions of precursor gases such as nitrogen oxides with volatile organic compounds that are mainly emitted from factories and car exhausts. Recently, ozone concentrations have rapidly increased in the developing Asian countries. For example, during 1982–2003, ozone concentrations in Beijing, China increased from a daily maximum of approximately 80 μg m^−3^ (about 40 nL L^−1^, ppb) to 250 μg m^−3^ (125 ppb) (Shao et al. 2006). High concentrations of ozone have also been observed frequently at an agricultural site in the Yangtze River Delta, China, with the highest values of daily 1-h maximum and monthly 7-h mean being 144 and 67 ppb, respectively, during 2007–2011 (Tang et al. 2013). In the 2020s under “Current legislation” scenario, which incorporates expected economic development and planned emission controls in individual countries, the annual average ozone levels showed maximum increases of 8–12 ppb in India, Pakistan, Bangladesh, China, and Southeast Asia comparing with the 1990s (Dentener et al. 2005).

Elevated ozone concentrations reduce the growth and yield of crop plants, including rice (*Oryza sativa* L.), which is the most important food crop in Asia (Kobayashi et al. 1995; Yonekura et al. 2005; Sawada and Kohno 2009). It is becoming clear that ozone influences not only grain yield but also grain quality of rice by increasing protein and reducing starch concentrations and absolute amounts of nutrient elements (Frei et al. 2012; Huang et al. 2012; Zheng et al. 2013). Moreover, elevated ozone increases the proportion of chalky grain, which is undesirable to the majority of consumers in the Far East (Wang et al. 2012, 2014). Wang et al. (2012) pointed out that the premature senescence of rice under ozone stress will conduce to incomplete filling of kernels, resulting in more kernels with a chalky appearance. However, the biochemical mechanisms responsible for ozone-induced changes in the components or appearance quality of rice kernels are poorly understood. It is noteworthy that deterioration in the grain quality occurs at a relatively low ozone level, even one not sufficient to reduce grain yield. Thus, impairment of rice grain quality by elevation of tropospheric ozone concentration is of greater concern than a decline in rice yield, especially in East Asia.

Recently, the grain quality of rice in Japan has often been impaired by high temperatures during the grain-filling period. The chalky phenotype of rice kernels is a typical symptom found in high-temperature ripened plants. Detailed observation has shown that the chalky region of a rice kernel contains many small amyloplasts containing small, single starch granules and shows numerous interspaces among the amyloplasts (Zakaria et al. 2002). Yamakawa et al. (2007) reported that transcription of genes for the biosynthesis of starch was suppressed and expression of genes for starch-consuming enzymes was induced by high temperature. Thus, inhibition of starch accumulation in kernels occurs during ripening under high temperature and results in chalkiness of the rice grain. However, the effect of elevated ozone concentration on starch accumulation in cereal kernels has been little reported. To our knowledge, only one study has measured starch synthesis enzyme activities in ozone-exposed wheat (Zhang et al. 2013). Thus, study of the effects of ozone elevation on rice quality is needed.

In this study, we analyzed the appearance quality and starch composition in the major Japanese rice cultivar “Koshihikari” (*japonica*) and the traditional Indian cultivar “Kasalath” (*indica*) under elevated ozone concentrations to clarify the mechanism of ozone-induced chalky kernel formation and irregular starch accumulation in rice kernels.

## Results

### Effect of Ozone on Grain Yield and Quality in Two Rice Cultivars

Grain yields were lowered to 4.2 and 5.2 % in “Koshihikari” and “Kasalath”, respectively, in plants grown under elevated ozone from that in plants grown under ambient air, although the difference was not statistically significant (Table 1). With ozone exposure, the filled kernel percentage was significantly decreased in both cultivars (*P* < 0.05). Thousands-grain weight was significantly reduced under elevated ozone only in “Koshihikari” (*P* < 0.001).

**Table 1:** Harvest parameters of two rice cultivars grown under AA and O_3_

Cultivars		Panicle number (/plant)	Total number of kernel (/plant)	Number of filled kernel (/plant)	Filled kernel percentage (%)	1000-grain weight (g)	Grain yield ^a^ (g/plant)	Relative yield (%)
Kos	AA	9.0 ± 0.9	642 ± 89.5	587 ± 83.2	91.2 ± 0.9	24.6 ± 0.2	14.4 ± 2.1	100.0
	O_3_	8.5 ± 0.5	670 ± 36.7	591 ± 33.2	88.2 ± 1.0	23.4 ± 0.1	13.8 ± 0.8	95.8
		ns	ns	ns	*	***	ns	
Kas	AA	9.1 ± 0.6	1234 ± 78.7	1175 ± 75.1	95.2 ± 0.4	17.9 ± 0.1	21.0 ± 1.4	100.0
	O_3_	7.8 ± 0.5	1218 ± 67.1	1134 ± 61.5	93.1 ± 0.3	17.6 ± 0.1	19.9 ± 1.0	94.8
		ns	ns	ns	***	ns	ns	

Values show mean ± SE (*n* = 12). ^a^grain yield was determined as rough rice grain weight in this report

Asterisks denote significant differences between ambient air (AA) and elevated ozone (O_3_) treatment (Student’s *t*-test, *P < 0.05, ***P < 0.001). *ns* not significant, *Kos* Koshihikari, *Kas* Kasalath

**Fig. 1:**
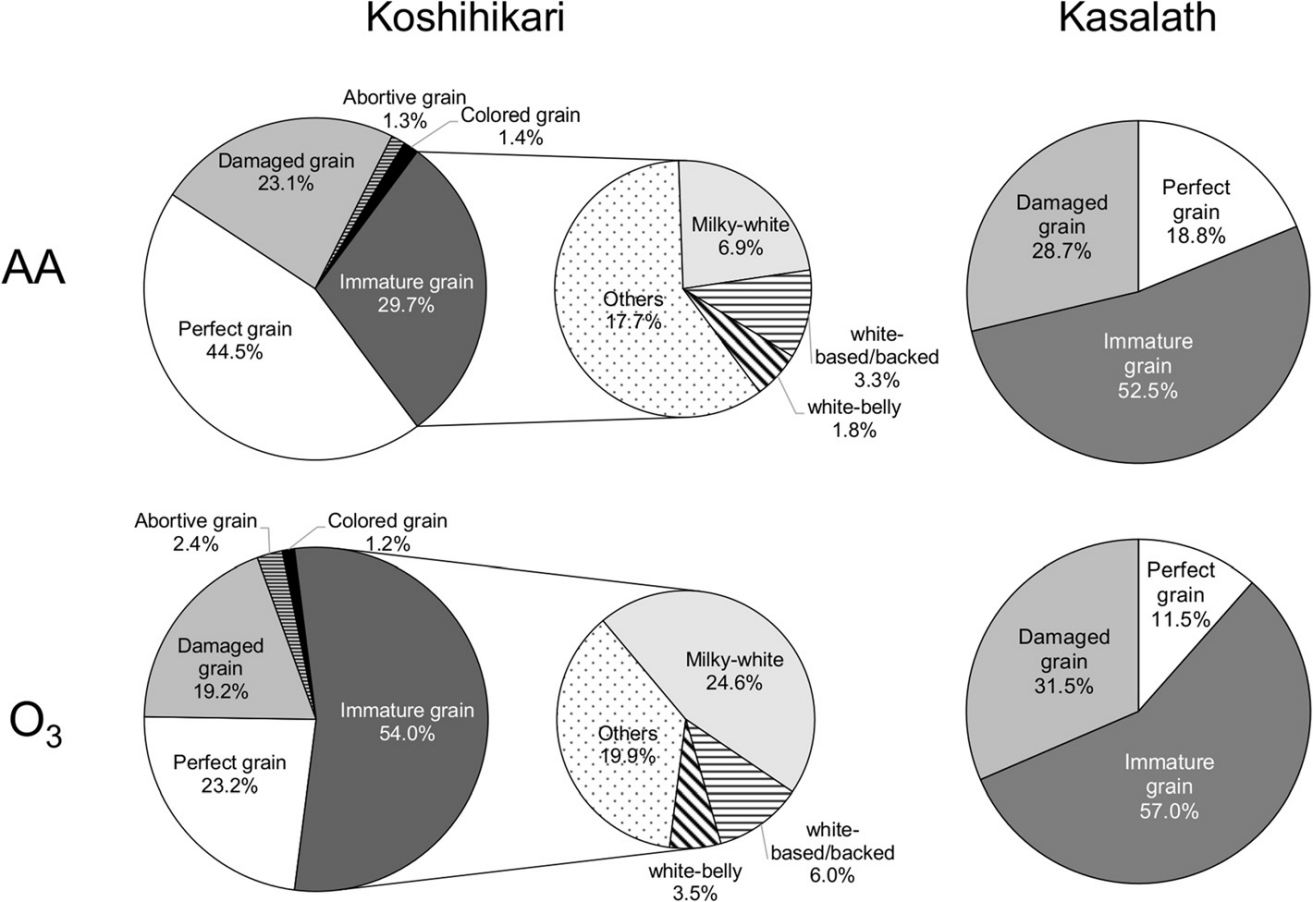
Appearance quality of dehulled kernels. The proportions of perfect (translucent), immature, damaged, abortive, and colored kernels were determined using a grain image analyzer, ES-1000 (Shizuoka Seiki) for “Koshihikari”. Immature kernels were further classified into milky-white, white-belly, and white-based/backed kernels with the ES-1000. The proportions of perfect, immature, and damaged kernels were determined by visual inspection in “Kasalath”. *AA* ambient air, *O*_*3*_ elevated ozone

The appearance of ozone-treated “Koshihikari” kernels was severely chalky (Additional file 1). Imaging analysis data for rice kernels showed that the proportion of immature kernels in “Koshihikari” increased with ozone treatment by 24 %, a value equal to the rate of decrease in perfect grain (Fig. 1). Among the immature kernels, “milky-white kernels,” which develop a chalky region at the center of the endosperm, were increased substantially in “Koshihikari” with ozone elevation. The proportion of “white-based/backed kernels” was also significantly higher in the kernels harvested from ozone-exposed rice (*P* < 0.05). To evaluate the grain quality of “Kasalath,” kernels were classified into the following categories by visual inspection: perfect, immature, or damaged kernels. In “Kasalath,” most of kernels were classified into immature or damaged without ozone treatment, and no significant difference in the proportion of kernel types was observed between elevated ozone and ambient air (Fig. 1 and Additional file 1).

### Appearance Traits of Kernels and Starch Granules in Ozone-Exposed “Koshihikari”

Grain shape dimensions in “Koshihikari” (average length, width, and thickness for each sample) were measured with a grain image analyzer (Table 2). Kernels from rice grown under elevated ozone were significantly shorter and thinner than those grown under ambient air (*P* < 0.01), although the average kernel width was not affected by ozone treatment. The average kernel density was significantly decreased in ozone-treated rice because the decrease in the average kernel weight (Table 1, 95.3 % of AA) was greater than the decrease in the average kernel volume.

**Table 2:** Size of rice kernels in “Koshihikari” grown under AA and O_3_

	Length (mm)	Width (mm)	Thickness (mm)	Volume (mm^3^)	Density (mg/mm^3^)
AA	4.96 ± 0.02	2.89 ± 0.01	1.93 ± 0.01	14.51 ± 0.14	1.69 ± 0.01
O_3_	4.89 ± 0.02	2.89 ± 0.01	1.90 ± 0.01	14.06 ± 0.12	1.66 ± 0.01
% of AA	98.7 **	100^ns^	98.2 **	96.9 *	98.4 *

Values show mean ± SE (*n* = 12). Asterisks denote significant differences between ambient air (AA) and elevated ozone (O_3_) treatment (Student’s *t*-test, *P < 0.05, **P < 0.01). *ns* not significant

**Fig. 2:**
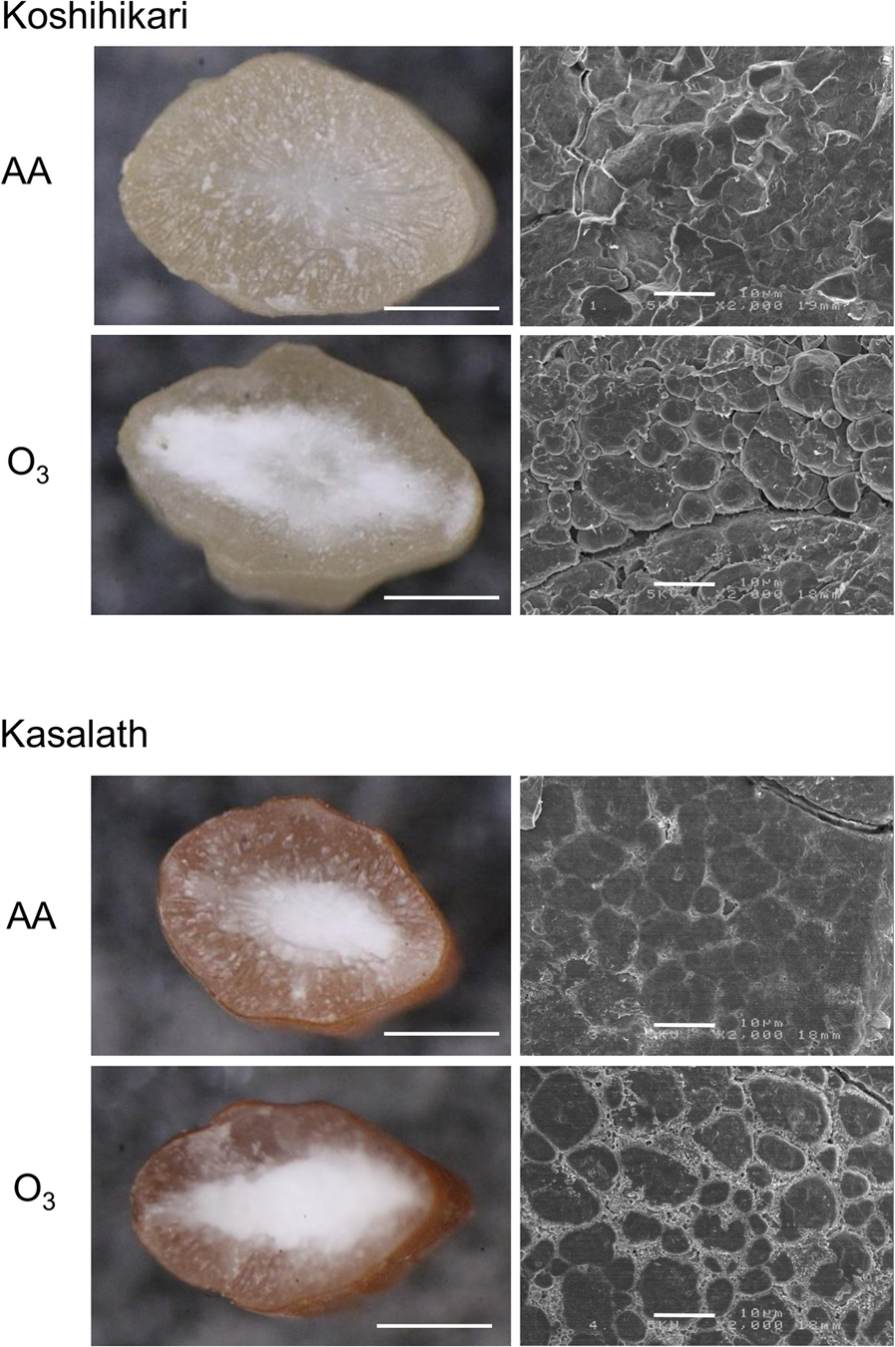
Transverse sections of kernels grown under AA and O_3_ in “Koshihikari” and “Kasalath”. Stereoscopic microscope images are shown on the left and scanning electron microscope (SEM) images are shown on the *right. Bars* in stereoscopic images, 10 mm; *bars* in SEM images, 10 μm. *AA* ambient air, *O*_*3*_ elevated ozone

Given that the reduction in grain density suggested that starch accumulation in the endosperm was inhibited by elevated ozone levels, we observed a transverse section of the rice kernels using visual and scanning electron microscopy (SEM) (Fig. 2). As indicated by the appearance data shown in Fig. 1, ozone induced chalky kernels in “Koshihikari”. SEM observation showed that translucent kernels of “Koshihikari” ripened under ambient air were filled with densely packed and similar sized granules with sharp edges. In contrast, the endosperm starch granules of “Koshihikari” ripened under elevated ozone conditions were loosely packed with large air spaces and contained irregular rounded granules. In “Kasalath”, a small chalky region was observed at the center of kernels from plants grown under ambient air, and the chalky region expanded to the kernel edge with ozone-exposure. Detailed observation using SEM showed that large starch granules were surrounded by a number of small and irregularly shaped starch granules in “Kasalath” kernels ripened under elevated ozone levels. Although the interspaces among large starch granules were filled with small starch granules, there were many small air spaces among small starch granules. A similar tendency was observed in the endosperm in “Kasalath” grown under ambient air, although fewer voids among large starch granules were detected than those in kernels grown under elevated ozone.

### Alterations of the Starch Component and Amylopectin Fine Structure in Ozone-Exposed “Koshihikari” and “Kasalath”

**Fig. 3:**
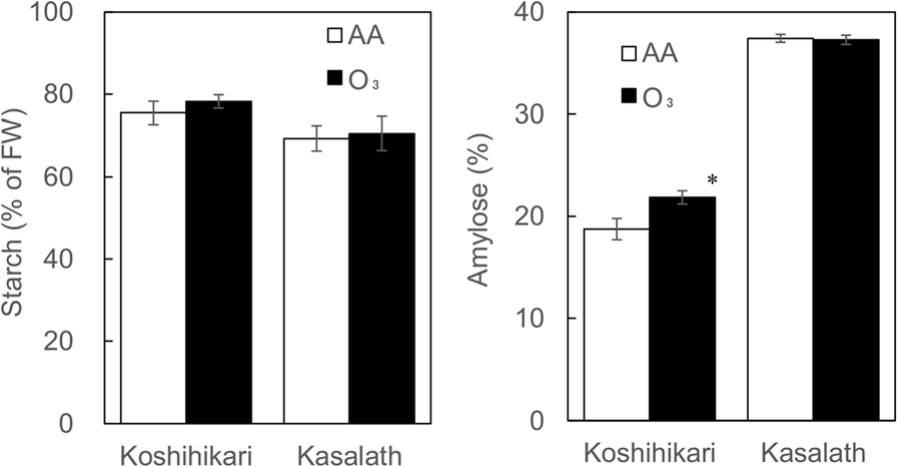
Effects of elevated ozone on starch and amylose content in “Koshihikari” and “Kasalath”. Values are mean ± SE (*n* = 4). *Asterisk* indicates a significant difference between AA and O_3_ treatment according to Student’s *t*-test (*P* < 0.05). *AA* ambient air, *O*_*3*_ elevated ozone

SEM observation showed that elevated ozone affected starch accumulation in the endosperm of “Koshihikari” kernels. We accordingly investigated the effects of elevated ozone on starch and amylose contents of kernels in “Koshihikari” and “Kasalath” (Fig. 3). Content of starch was not affected by ozone in kernels of both cultivars. Amylose content was increased in “Koshihikari” but unchanged in “Kasalath” kernels grown under elevated ozone levels.

**Fig. 4:**
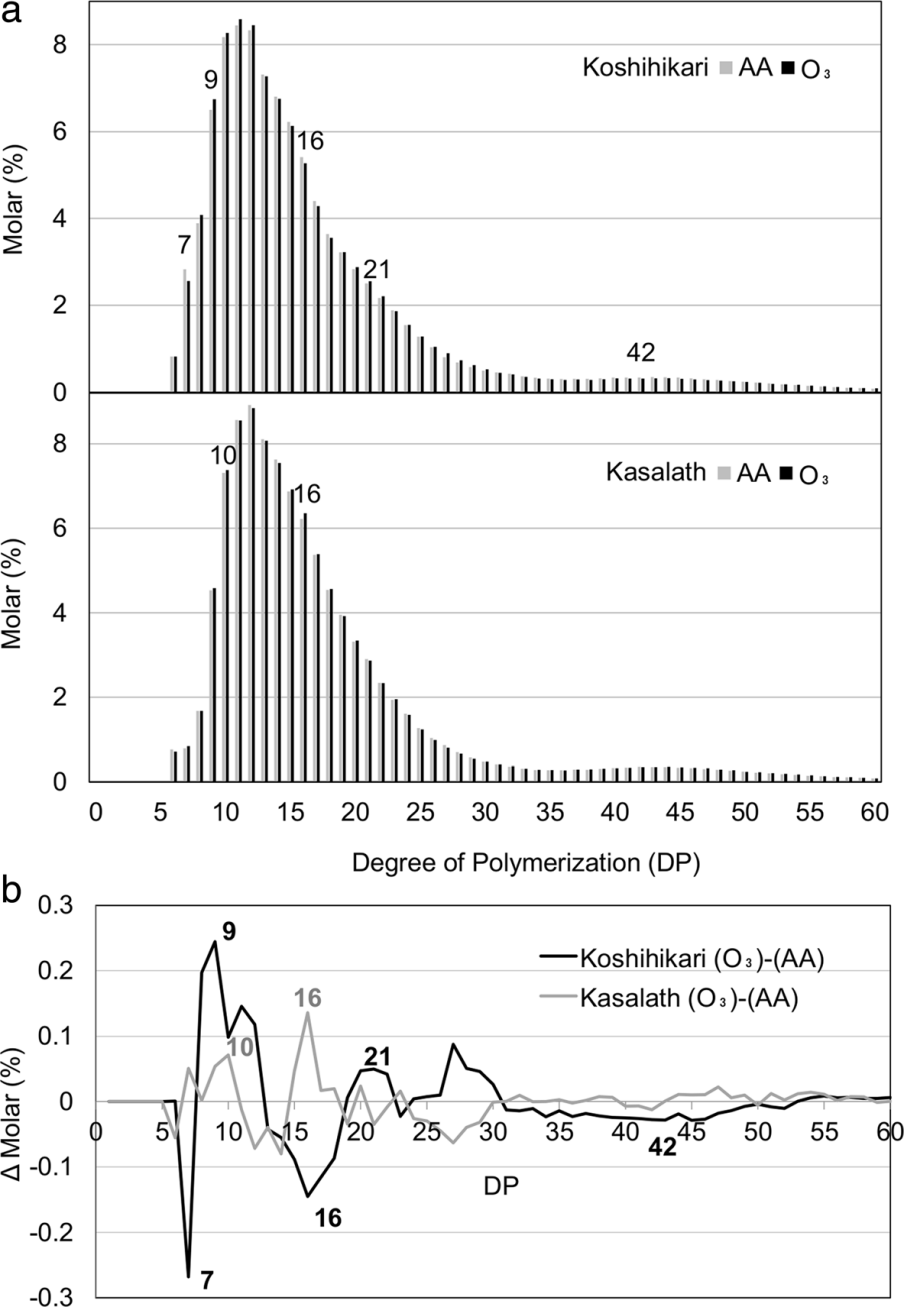
Chain-length distribution patterns of endosperm amylopectin in kernels grown under AA and O_3_. (**a**: *top*) “Koshihikari” and (**a**: *bottom*) “Kasalath”. Debranched amylopectin extracted from AA-grown plants (*gray bars*) or O_3_ (*black bars*) was analyzed using the FCEP method, and the relative peak area of the chromatogram is shown for individual DP. **b** Comparison of differences in the chain-length distribution pattern of amylopectin (delta molar %) between “Koshihikari” and “Kasalath”. The difference in relative peak area in B between AA and O_3_ is shown. The *numbers* in the plots are DP values. *AA* ambient air, *O*_*3*_ elevated ozone

To evaluate the effects of elevated ozone on starch components in more detail, the side-chain length distribution of amylopectin was determined by the fluorescence capillary electrophoresis (FCEP) method (Fig. 4). In “Koshihikari” kernels, elevated ozone levels resulted in increased numbers of chains with a degree of polymerization (DP) 8–12, 19–22, and 25–30 and decreased numbers of chains with DP 7, 13–18, and 31–52. The alteration pattern in length distribution of amylopectin side chains in ozone-exposed “Kasalath” kernels differed from that in “Koshihikari” kernels. In “Kasalath” kernels, chains with DP 7–10 and 15–18 were increased and chains with DP 12–14 and 24–29 were decreased.

### Expression Levels of Genes Involved in Starch Synthase in Ozone-Exposed Rice Cultivars

**Fig. 5:**
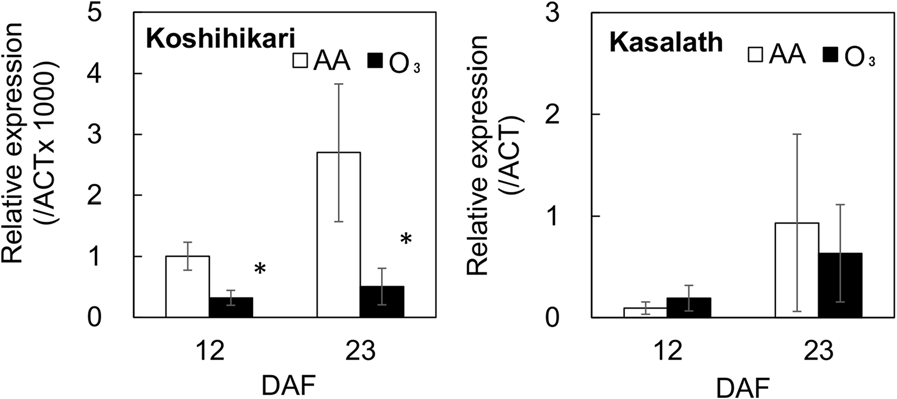
Relative expression levels of *SSIIIa* transcript in rice caryopses grown under AA and O_3_. Values are mean ± SE (*n* = 3). *Asterisk* indicates a significant difference between AA and O_3_ treatment by Student’s *t*-test (*P* < 0.05). *DAF* day after flowering, *AA* ambient air, *O*_*3*_ elevated ozone

Given that the content of long chains of amylopectin with DP > 30 was reduced in “Koshihikari” kernels under elevated ozone levels from those in kernels grown under ambient air, we evaluated the expression in rice caryopses of the *STARCH SYNTHASE (SS) IIIa* gene, which plays an important role in generating long chains of amylopectin, and of other genes involved in starch synthesis (*SSI* and *GRANULE BOUND (GB) SSI*) (Fig. 5 and Additional file 2). On 12 day after flowering (DAF), the mRNA level of *SSIIIa* in ozone-exposed “Koshihikari” was reduced to 30 % of that of plants grown under ambient air. The reduction in the gene expression in “Koshihikari” by ozone was increased to 19 % at 23 DAF. The *SSIIIa* transcript level in “Kasalath” was higher than that in “Koshihikari”, but was not affected by ozone treatment. The levels of *SSI* and *GBSSI* transcripts were not changed significantly by ozone treatment in either cultivar (Additional file 2).

### Analysis of the Physicochemical Properties of Rice Kernels in Ozone-Exposed “Koshihikari” and “Kasalath”

To evaluate the effect of the alteration of starch structure on physicochemical properties, we analyzed the starch pasting properties of ground kernels using a rapid viscoanalyzer (RVA; Table 3 and Additional file 3). Maximum viscosity increased slightly in “Koshihikari” grown under elevated ozone (*P* < 0.1). The property of breakdown showed contrasting responses to ozone in the two cultivars: the breakdown value was increased significantly in ozone-exposed “Koshihikari” but decreased in “Kasalath” (*P* < 0.05). The setback value decreased and peak time increased significantly in ozone-treated “Kasalath” (*P* < 0.05). The pasting temperature increased slightly in kernels of ozone-treated “Kasalath” (*P* < 0.1).

**Table 3:** Pasting properties by RVA of the flours in two rice cultivars grown under AA and O_3_

		Maximum viscosity (cP)	Minumum viscosity (cP)	Breakdown (cP)	Final viscosity (cP)	Setback (cP)	Peak Time (min)	Pasting Temp (°C)
Kos	AA	3511 ± 39	1636 ± 43	1875 ± 12	2577 ± 37	941 ± 13	6.24 ± 0.04	66.4 ± 0.03
	O_3_	3605 ± 15	1633 ± 23	1972 ± 21	2542 ± 22	909 ± 19	6.26 ± 0.01	66.4 ± 0.04
		*	ns	**	ns	ns	ns	ns
Kas	AA	2429 ± 53	1334 ± 42	1095 ± 12	2932 ± 54	1597 ± 14	6.12 ± 0.02	70.9 ± 0.25
	O_3_	2307 ± 41	1455 ± 40	852 ± 19	2943 ± 44	1488 ± 11	6.33 ± 0.03	71.8 ± 0.15
		ns	ns	***	ns	***	***	*

Values show mean ± SE (*n* = 3). Asterisks denote significant differences between ambient air (AA) and elevated ozone (O_3_) treatment (Student’s *t*-test, *P < 0.1, **P < 0.05 and ***P < 0.01). *ns* not significant, *Kos* Koshihikari, *Kas* Kasalath

## Discussion

The appearance quality of rice kernels is important, given that chalky kernels are readily broken during milling, reducing the market price. In this study, we showed that marked deterioration in appearance quality in the *japonica* cultivar “Koshihikari” was induced by an elevated level of ozone that did not lead to grain yield reduction (Table 1, Fig. 1). In contrast, no significant change was detected in the proportion of chalky kernels in “Kasalath” grown under elevated ozone, suggesting that the appearance quality of “Kasalath” is insensitive to elevated ozone.

The deterioration of appearance quality found in ozone-exposed “Koshihikari” kernels occurred as the result of the marked increase in milky-white kernels under elevated ozone (Figs. 1 and 2). The chalky appearance is derived from a change in light refraction resulting from the occurrence of numerous air spaces between loosely packed starch granules (Tashiro and Wardlaw 1991). The determinations of kernel weight and kernel dimensions shown in Tables 1 and 2 suggest a lower density of kernels in ozone-exposed “Koshihikari,” indicating an increase in air spaces in the endosperm.

To clarify the internal structure of the rice endosperm, transverse sections of rice kernels were observed with SEM (Fig. 2). Endosperm starch of “Koshihikari” grown under elevated ozone contained small and irregular rounded granules resulted in the creation of large air spaces between themselves. In contrast, the numbers of air spaces in “Kasalath” endosperm were fewer than those in “Koshihikari” under elevated ozone. This is the first report of ozone-induced abnormal accumulation of starch in rice kernels revealed by SEM observation. Although alteration of amyloplast structure observed in ozone-exposed kernels was similar to that in kernels ripened under high temperature, small holes in the surface of starch granules, found in kernels ripened under high temperature, were not observed in kernels exposed to ozone (Tashiro and Wardlaw 1991; Zakaria et al. 2002). This finding indicates that the underlying mechanism of the deterioration of appearance quality of rice grain differs between ozone exposure and high-temperature stress.

Previous study showed that grain chalkiness is occurred as a consequence of alterations in amylopectin structure (Yamakawa et al. 2007). We found that abnormal starch accumulation was induced by elevated ozone in “Koshihikari” and “Kasalath”. In the *japonica* cultivar “Koshihikari”, amylose content increased significantly with elevated ozone, although starch content was not affected by ozone (Fig. 3). Distribution analysis of amylopectin chain-length showed a decrease in long side chains of DP > 30 as well as alterations of short side chains of DP < 20 in the kernels of ozone-treated “Koshihikari”. In contrast, amylose content was not changed by ozone and amylopectin chain length tended to increase in both short and long side-chains in kernels of the *indica* cultivar “Kasalath”. In previous reports, elevated ozone decreased or did not change the amylose content in hybrid *indica* rice grain (Wang et al. 2012, 2014; Zheng et al. 2013), but there are no reports of the distribution pattern of amylopectin chain length in kernels ripened under elevated ozone. The effect of ozone elevation on starch composition appears to differ between *japonica* and *indica* rice cultivars. Interestingly, high-temperature treatment reduced the amylose content of *japonica* rice, a response differing from that to the ozone-induced change in the kernel amylose content. Moreover, rising temperature during the grain-ripening period resulted in a decrease in short side chains of DP 10–19 and an increase of longer side chains of DP 21–32 and > 41 (Yamakawa et al. 2007), suggesting that the pattern of alteration in distribution of amylopectin chain length under high temperature differs from that under elevated ozone. Taken together, our results suggest that chalky kernels found in ozone-exposed “Koshihikari” are generated by a novel mechanism that differs from that of the induction of chalky kernels under high temperature.

The chain-length distribution pattern of amylopectin in kernels from ozone-exposed “Koshihikari” was similar to that in kernels from *SSIIIa*-deficient rice mutants (Fujita et al. 2007). We accordingly evaluated *SSIIIa* expression in “Koshihikari” and “Kasalath” caryopses during the grain-filling period (Fig. 5). The transcript level of *SSIIIa* was decreased by ozone in “Koshihikari”, whereas no such decrease was detected in “Kasalath”. Previous studies showed that the *ssIIIa* mutants result in a chalky interior appearance in kernels and an irregular shape of starch granules caused by insufficient crystallization of starch (Fujita et al. 2007; Ryoo et al. 2007). Furthermore, the authors suggested that SSIIIa plays an important role in the elongation of long-B chains (DP > 30) connecting the amylopectin cluster. We thus propose that the chalky phenotype in ozone-exposed “Koshihikari” kernel is attributed to loosely packed starch granules, which is caused by the reduction of long chains of amylopectin resulting from the decrease in the expression of *SSIIIa*. On the other hand, “Kasalath” showed high percentage of chalky grain (Fig. 1), and the *SSIIIa* transcript levels in the cultivar were higher than those in “Koshihikari” with or without ozone exposure (Fig. 5). Xu et al. (2015) showed that *Indica* rice was inferior in the appearance quality compared with *japonica* rice regardless of their growth conditions, suggesting that the inherent high chalky grain in “Kasalath” is independent from the expression levels of *SSIIIa*. Although further studies, such as expression analysis of other genes involved in starch synthesis or metabolism, are needed to determine the role of *SSIIIa* on the alteration of starch structure under elevated ozone, our results indicate the presence of a novel mechanism of ozone-induced deterioration of the appearance quality in *japonica* rice.

The viscosities of pasting starch of rice kernels were analyzed to evaluate the effect of elevated ozone on eating quality (Table 3 and Additional file 3). Chamura et al. (1979) reported a negative correlation between amylose content and maximum viscosity among rice cultivars. In “Koshihikari”, both the amylose content and maximum viscosity were increased by ozone treatment, suggesting that the increase of amylose content alone could not account for the reduction in eating quality by ozone. Igarashi (2010) showed that the molar ratio of short/long unit-chains of amylopectin decreased when the temperature during grain filling was high, resulting in a decrease in breakdown and increases in both minimum viscosity and pasting temperature. In the present study, the molar ratio of shorter unit-chains (DP ≤ 30) to long unit-chains (DP > 30) of amylopectin was increased in ozone-exposed “Koshihikari” kernels (11.0 ± 0.2 under ambient air and 11.8 ± 0.2 under elevated ozone), whereas those in “Kasalath” kernels were decreased (12.7 ± 0.2 under ambient air and 11.5 ± 0.2 under elevated ozone). The correlations between the change in the molar ratio of amylopectin and the change of pasting properties occurring under ozone elevation are consistent with those reported by Igarashi (2010). This result suggests that the chain distribution of amylopectin affects the alteration of pasting properties rather than amylose content in rice kernels under elevated ozone concentration.

In general, higher values of maximum viscosity and breakdown and lower values of setback are closely associated with better palatability of rice (Suzuki 1979; Chikubu et al. 1983). Elevated ozone significantly reduced the appearance quality of “Koshihikari” kernels, but may have improved its eating quality by increasing the values of the maximum viscosity and breakdown. In contrast, “Kasalath” kernels, which did not change in appearance quality under elevated ozone levels, showed reduced breakdown values and increased pasting temperature, suggesting detrimental effects on eating quality. However, eating quality should be evaluated by further analysis of other components, such as protein and amino acid content, and of physicochemical properties such as texture and appearance of cooked rice (Suzuki et al. 2006), that could also affect eating quality.

## Conclusions

The *japonica* cultivar “Koshihikari” showed deteriorations in appearance quality under ozone stress, which occurred at a level of ozone insufficient to cause grain yield reduction. The deterioration in grain quality was induced by abnormal starch accumulation under elevated ozone levels as well as by high-temperature stress. However, the alteration patterns of amylose and amylopectin in ozone-exposed “Koshihikari” were similar to those in *SSIIIa*-deficient rice mutants rather than those in kernels ripened under high-temperature. Furthermore, the *SSIIIa* transcript in “Koshihikari” caryopses was suppressed by elevated ozone levels. Thus, the increase in chalky kernels in “Koshihikari” may result from the decreased expression, under elevated ozone, of *SSIIIa* involved in amylopectin side-chain elongation.

## Methods

### Plant Materials and Ozone Treatment

Seeds of rice cultivars “Koshihikari” (a *japonica* cultivar) and “Kasalath” (an *indica* cultivar) were sown in seedling boxes and grown for 3 weeks in a glasshouse under ambient air. The seedlings were transplanted into pots (0.05-m^2^ surface area and 0.015-m^3^ volume) (four pots of each cultivar) on June 6 and grown until September 30, 2014 in commercial-type vinyl greenhouses located in the experimental field of the National Institute for Environmental Studies (Tsukuba, Ibaraki). Fertilizer (N:P:K 8:8:5) was supplied at 150 g m^−2^ before transplanting. The dimensions of the exposure chamber were 3.15-m width, 1.8-m height, and 16.2-m length and the long axes of the chambers were in north–south orientation. Rice plants were exposed daily to ambient air (AA) or ozone-supplemented air (O_3_) through air ducts from June 25 to September 19, 2014. The experiment was arranged in a randomized complete block design within the whole plot with four replicates of AA and O_3_ chambers. Ozone was generated using an electrical discharge ozone generator (Model SG-1, Dylec Inc., Ibaraki, Japan) from industrial-grade oxygen of 99.5 % purity as the source gas. Concentrations of ozone were continuously monitored at three points in each greenhouse at 10-min intervals using a UV absorption ozone analyzer (Model 1210-6, Dylec Inc.). Daily mean ozone concentrations for 7 h (10:00–17:00 Japanese Standard Time) were 36 (AA) and 71 (O_3_) ppb (Additional file 4). The average air temperature and relative humidity in the each greenhouse from June to September were 26.3 °C and 60.3 % (AA), and 26.6 °C and 59.9 % (O_3_), respectively. The average of air temperature, relative humidity, and light intensity (photosynthetically active radiation) in both glasshouses were not significantly different throughout the growing period.

### Measurement of Yield and Grain Quality Traits

Rice cultivars were harvested on September 30, 2014. All yield data were determined by measurements of 12 individual plants for each treatment. Kernels were separated from panicles and categorized into two groups: filled grain and unfilled grain, using an automatic seed-sorting machine (FV-459A, Fujiwara Seisakusho KK, Tokyo, Japan). The filled kernels (rough rice) were weighed to determine the grain yield. Filled kernels in “Koshihikari” were unhusked and appearance quality was determined by a rice grain image analyzer (ES-1000; Shizuoka Seiki Co., Ltd., Shizuoka, Japan) that classified kernels into perfect, immature, damaged, abortive, and colored kernels. Immature kernels, is a term according to the official inspection standard of the Japan Food Agency, means “insufficient filling” kernels, were further classified into milky-white, white-belly, and white-based/white-back kernels. Kernel physical attributes (kernel length, width, and thickness) were measured using another rice image analyzer (Satake Engineering Corp., Tokyo, Japan). Kernel volume (*V*, mm^3^) was calculated as follows:

$$ V=4/3\times L/2\times D/2\times T/2\times \pi $$

where *L*, *D*, and *T* is the length (mm), width (mm), and thickness (mm) of the kernels, respectively. Bulk density was calculated by dividing kernel weight (mg, the date are presented in Table 1) by kernel volume (mm^3^) (Fukumori and Mishima 2002).

To determine the grain quality of “Kasalath,” 100 unhusked kernels were categorized by visual inspection as perfect, immature, or damaged kernels, because *indica*-type kernels were not adapted to analysis by the grain image analyzer.

For measurement of amylose and starch content, unhusked brown rice kernels were polished to remove the embryo and pericarp with a test mill (Pearlest; Kett Electric Laboratory, Tokyo, Japan), after which these kernels were ground into powder with a food mill (Milser, IFM-800DG, Iwatani Corporation, Tokyo, Japan).

### Scanning Electron Microscopy (SEM)

Brown rice kernels were cut transversely with a razor blade and the cracked surfaces were coated with osmium metal. The surfaces were viewed with a JSM-6320F scanning electron microscope (JEOL Ltd., Tokyo, Japan) at an acceleration voltage of 5 kV.

### Determination of Amylose Content in Kernels

Apparent amylose content was measured by an iodine colorimetric method (Yamakawa et al. 2007). Twenty milligrams of polished rice powder was gelatinized by treatment with 0.1 mL of 95 % ethanol and 0.9 mL of 1 M NaOH and was kept for 10 min in boiling water. After the addition of 5 mL of distilled water, the solution was homogenized and filled to 10 mL with distilled water. An aliquot (1 mL) of the solution was added to 0.2 mL of 1 M acetic acid, 0.2 mL of 0.2 % (*w/v*) I_2_, 2 % (*w/v*) KI, and 8.6 mL of distilled water.

After incubation at 27 °C for 20 min, A620 was measured using a SmartSpec Plus spectrophotometer (Bio-Rad Laboratories Inc., Hercules, CA, USA). The apparent amylose concentration was estimated by the method of Juliano (1971) from the base calibration line, which was obtained from the absorbance values by changing the ratio of potato amylose (type III, Sigma Chemical Co., St. Louis, MO, USA) and waxy rice powder in the iodine solution.

### Determination of Starch Content in Kernels

Twenty milligrams of polished rice powder was homogenized with 4 mL of dimethyl sulfoxide and 1 mL of HCl (8 mol L^−1^) at 60 °C for 30 min. The reaction solution was adjusted to pH 4–5, and the starch concentration was determined using the enzymatic method (F-kit starch, Roche Diagnostics, Mannheim, Germany).

### Determination of the Distribution of *α*-Glucan Chain Length of α-Polysaccharides

Chain length distribution analyses of α-glucan was performed according to the method of Fujita et al. (2012). Dehulled rice kernels (0.3–1.0 g) were ground with a mortar and pestle and the powder was suspended in 3–4 mL of distilled water. The suspension was centrifuged at 600 × *g* at 20 °C for 10 min. Three volumes of methanol were added to the supernatant, and the mixture was kept at 4 °C overnight. The precipitate was collected by centrifugation at 3000 × *g* at 4 °C for 10 min. The precipitate was washed by suspension in 2 mL of ice-cold methanol followed by centrifugation at 10,000 × *g* at 4 °C for 10 min. The sample was dried in a centrifugal vacuum evaporator. The chain length distributions of α-glucans from endosperm were analyzed using the FCEP method of O’Shea and Morell (1996) and Fujita et al. (2001) in a P/ACE MDQ Carbohydrate System (Beckman Coulter, Fullerton, CA, USA).

### Quantitative Polymerase Chain Reaction (PCR) Analysis

Total RNA was isolated from developing caryopses taken at 12 and 23 DAF of “Koshihikari” and “Kasalath” grown under AA and O_3_ conditions, using an RNeasy Plant Mini Kit (Qiagen, Valencia, CA, USA) following the manufacturer’s instructions. A 1-μg aliquot of total RNA was reverse-transcribed using random hexamers and ReverTra Ace® qPCR Master Mix with gDNA Remover (TOYOBO CO., LTD., Osaka, Japan) in a 20 μL reaction volume, and 2 μL of the reaction mixture was used for quantitative real-time PCR. Quantitative real-time PCR was performed in a LightCycler 480 System (Roche Applied Science, Mannheim, Germany) using KOD SYBR® qPCR Mix (TOYOBO) according to the manufacturer’s specifications. A fragment of cDNA was amplified with the PCR primers 5′-GCCTGCCCTGGACTACATTG-3′and 5′-GCAAACATATGTACACGGTTCTGG-3′ for *SSIIIa (GenBank accession no: AY100469)*, 5′-GGGCCTTCATGGATCAACC-3′ and 5′-CCGCTTCAAGCATCCTCATC -3′ for *SSI (GenBank accession no: D16202)*, and 5′-AACGTGGCTGCTCCTTGAA -3′ and 5′-TTGGCAATAAGCCACACACA -3′ for *GBSSI (GenBank accession no: X62134)*. As an internal standard for cDNA amounts, a fragment of actin 1 cDNA *(GenBank accession no: AB047313)* was amplified with PCR primers 5′-CTTCATAGGAATGGAAGCTGCGGGTA-3′ and 5′-CGACCACCTTGATCTTCATGCTGCTA-3′. The relative expression level of each gene was calculated by dividing the value of each gene by the value of the actin 1 signal. Three independent biological samples were used with the gene-specific primers.

### Pasting Properties

The pasting properties of rice flours were determined with a rapid viscoanalyzer (RVA) (RVA Rice Master, Newport Scientific, Sydney, Australia) by the method of Toyoshima et al. (1997). The properties were expressed as the starting gel point temperature, peak viscosity (maximum viscosity), breakdown viscosity, (the difference between peak and minimum viscosity), and setback viscosity (the difference between minimum and final viscosity).

### Statistics

Software (IBM SPSS Statistics version 22; IBM) was used for statistical analyses. To assess the statistical significance of treatment differences, *t*-tests (with *P* set at 0.05) were used. Statistical analyses were performed for individual plant data for yield, yield components, and quality, and for individual pot data for starch analysis.

## Additional files

Additional file 1:
**Visual images of dehulled kernels. Black and white arrowheads indicate severely chalky and almost translucent grains, respectively.** AA, ambient air; O_3_, elevated ozone. (TIF 4577 kb) Additional file 2:
**Relative expression levels of** ***SSI*** **and** ***GBSSI*** **transcript in rice caryopses grown under AA and O**_**3**_ **at day after flowering 12.** Values are mean ± SE (*n* = 3). AA, ambient air; O_3_, elevated ozone. (TIF 7760 kb) Additional file 3:
**Pasting properties of rice kernels of “Koshihikari” and “Kasalath”.** The viscosity value at each temperature point is the average of three replications. The thin line indicates the change in temperature during measurement with a RVA. AA, ambient air; O_3_, elevated ozone. (TIF 11376 kb) Additional file 4:
**Daily ozone concentrations for AA and O**_**3**_ **exposure chambers.** Values represent mean ozone concentration each hour of the day averaged from 25 June to 19 September in 2014. AA, ambient air; O_3_, elevated ozone. (TIF 9538 kb)

## Abbreviations

AA: ambient air
DAF: day after flowering
DP: degree of polymerization
SEM: scanning electron microscopy
SS: starch synthase
