# Supplementary figures and images for: Elevated Ozone Deteriorates Grain Quality of Japonica Rice cv. Koshihikari, Even if it Does Not Cause Yield Reduction
Source: Rice (N Y). 2016 Feb 24;9:7. doi: 10.1186/s12284-016-0079-4 (PMC4766164; doi:10.1186/s12284-016-0079-4)

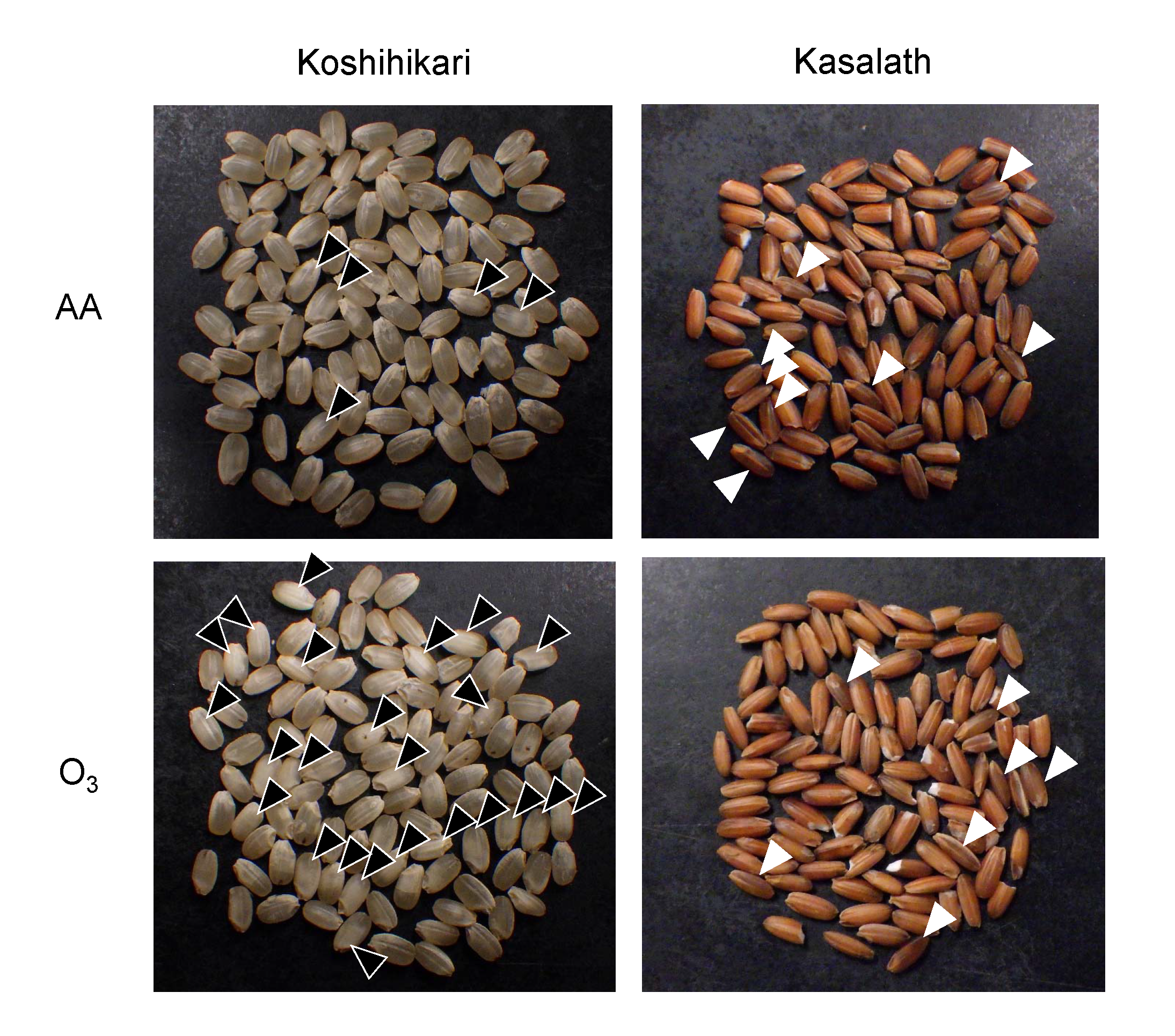

Supplement: Additional file 1: — Visual images of dehulled kernels. Black and white arrowheads indicate severely chalky and almost translucent grains, respectively. AA, ambient air; O3, elevated ozone. (TIF 4577 kb) [file 12284_2016_79_MOESM1_ESM.tif]

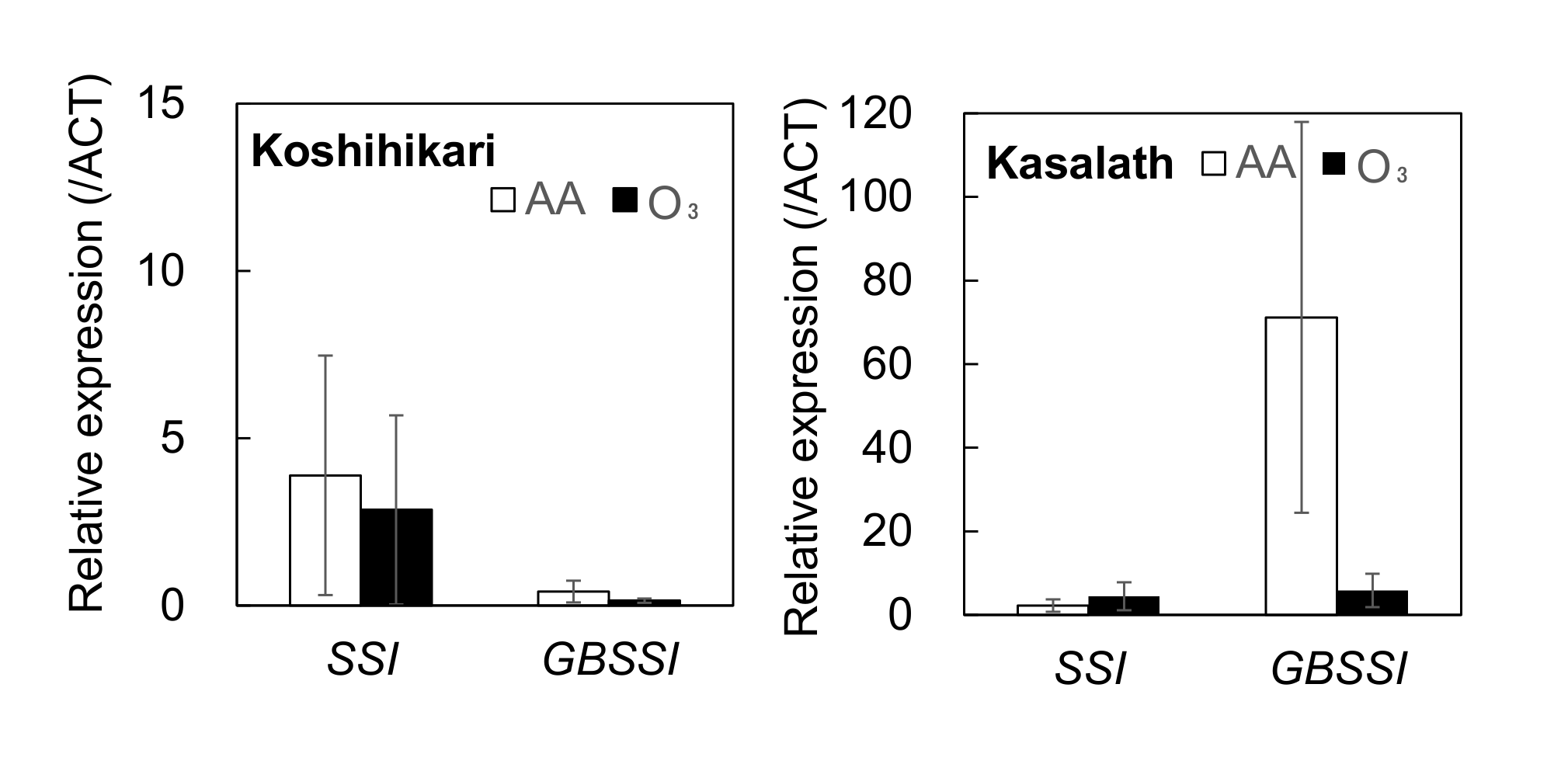

Supplement: Additional file 2: — Relative expression levels of SSI and GBSSI transcript in rice caryopses grown under AA and O 3 at day after flowering 12. Values are mean ± SE (n = 3). AA, ambient air; O3, elevated ozone. (TIF 7760 kb) [file 12284_2016_79_MOESM2_ESM.tif]

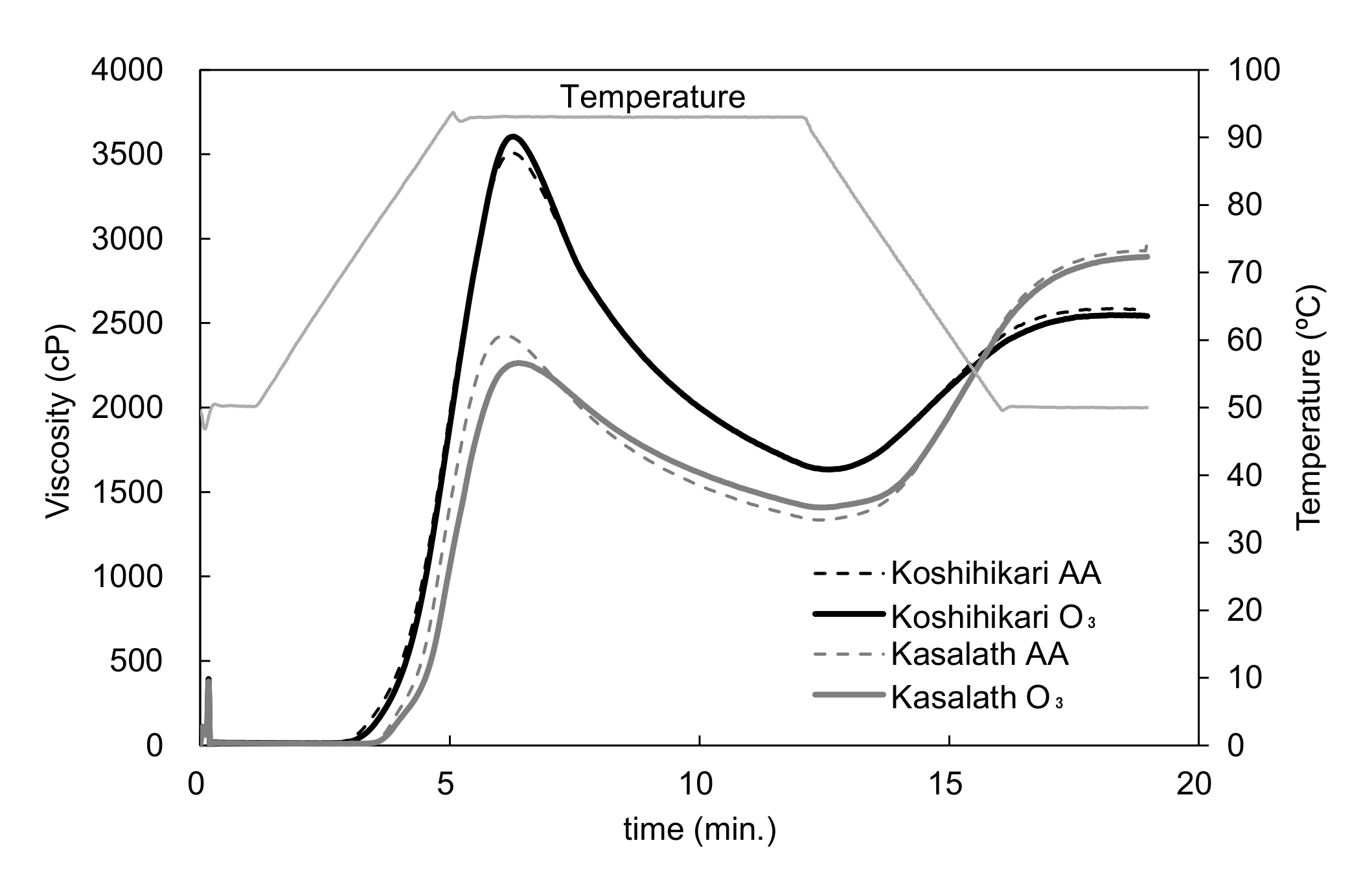

Supplement: Additional file 3: — Pasting properties of rice kernels of “Koshihikari” and “Kasalath”. The viscosity value at each temperature point is the average of three replications. The thin line indicates the change in temperature during measurement with a RVA. AA, ambient air; O3, elevated ozone. (TIF 11376 kb) [file 12284_2016_79_MOESM3_ESM.tif]

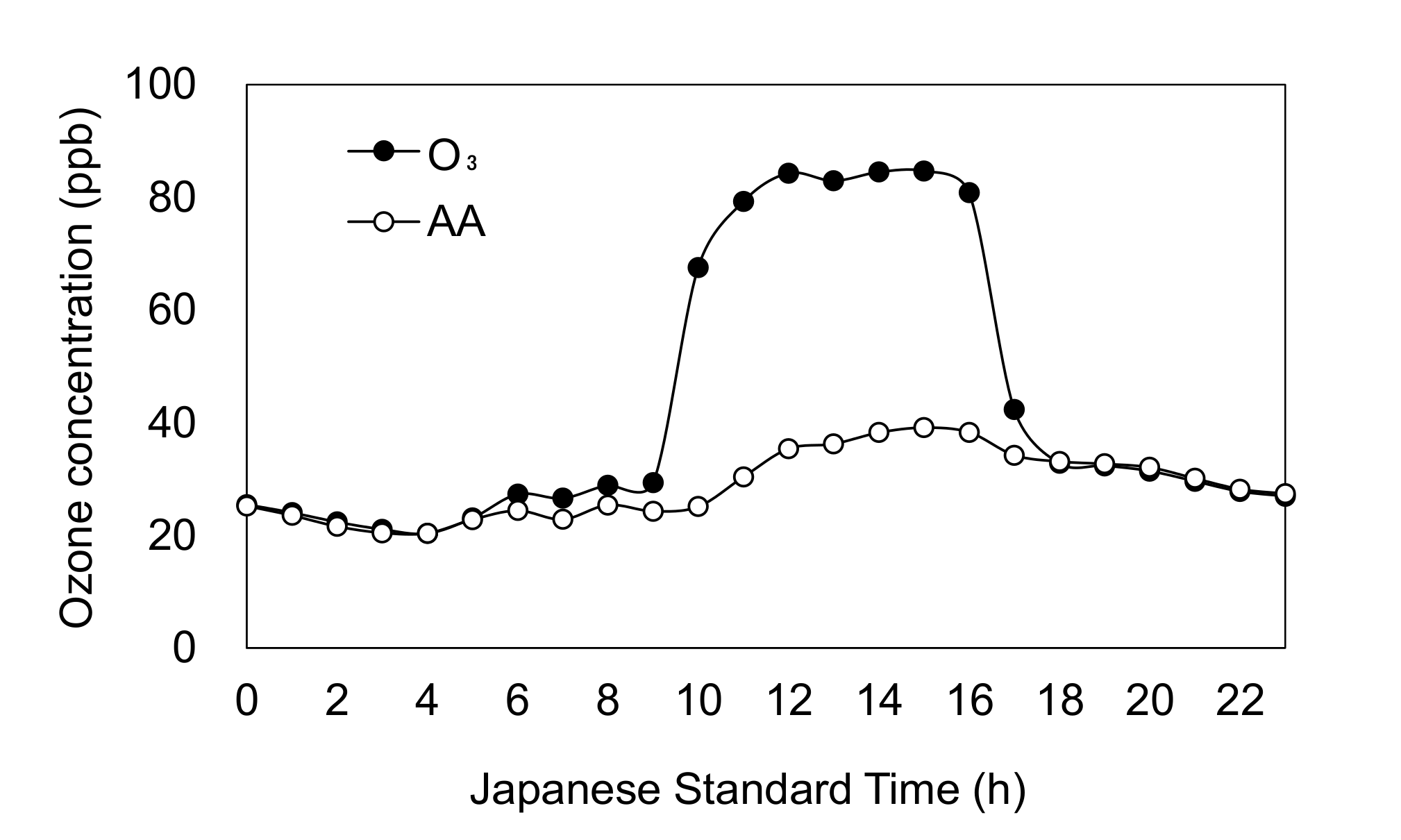

Supplement: Additional file 4: — Daily ozone concentrations for AA and O 3 exposure chambers. Values represent mean ozone concentration each hour of the day averaged from 25 June to 19 September in 2014. AA, ambient air; O3, elevated ozone. (TIF 9538 kb) [file 12284_2016_79_MOESM4_ESM.tif]
